# Organization and support as an essential part of group exercise programs for older people with dementia: an end-user interview study

**DOI:** 10.3389/fspor.2025.1526163

**Published:** 2025-07-31

**Authors:** Kristin Taraldsen, Arnhild J. Nygård, Elisabeth Boulton, Guro Grønningsæter, Marit H. Erland, Nina Waldenstrøm, Linda Johnsen, Gro G. Tangen, Randi Granbo

**Affiliations:** ^1^ Department of Rehabilitation Science and Health Technology, Faculty of Health Sciences, Oslo Metropolitan University (OsloMet), Oslo, Norway; ^2^ Department of Neuromedicine and Movement Science, Faculty of Medicine and Health, Norwegian University of Science and Technology (NTNU), Trondheim, Norway; ^3^ Age UK, London, United Kingdom; ^4^ Molde Municipality, Molde, Norway; ^5^ Byåsen Frivillighetssentral, Trondheim, Norway; ^6^ Unit for Physiotherapy Services, Trondheim Municipality, Trondheim, Norway; ^7^ Department of Physiotherapy, Nordre Follo Municipality, Nordre Follo, Norway; ^8^ Norwegian National Centre for Ageing and Health, Vestfold Hospital Trust, Tønsberg, Norway; ^9^ Department of Geriatric Medicine, Oslo University Hospital, Oslo, Norway

**Keywords:** dementia, exercise, health services for the aged, aged, aged 80 and over

## Abstract

**Introduction:**

Development of accessible group exercise sessions is warranted for home-dwelling older people with cognitive impairment or dementia. This study aims to explore the experiences of participants in a group exercise session organized with volunteers both as instructors and as those providing support for the participants.

**Methods:**

This qualitative descriptive study reports on a primary analysis of qualitative data collected through semistructured focus group interviews with 12 people with cognitive impairment or dementia at three group exercise sessions in three municipalities. Interviews were audio-recorded, transcribed, and analyzed using thematic analysis.

**Results:**

Participants expressed an overall satisfaction with the new group exercise sessions. We found four meaning units forming a chain of support. The participants highlighted support to be motivated enough to engage in the sessions, support by offering transportation, the exercise content, and the role of instructors. Overall, the participants expressed that these exercise sessions had become a social arena for them, and all wanted to continue.

**Discussion and conclusions:**

Exercise groups can be a meaningful and social arena for people with cognitive impairment or dementia, through careful organization with volunteers and minimal involvement from informal caregivers. This study underlines the need for properly organized activities, outside the home, to overcome the challenges associated with participation for this population.

## 1 Introduction

Dementia is one of the most common age-related diseases, with numbers projected to increase in Norway from 101,118 in 2020 to 236,789 by 2050 (1). Physical activity (PA) is recommended for everyone, as it is a well-known modifiable factor for health (2). People with various levels of cognitive impairment can benefit from exercise (3), and for people living with dementia, exercise may improve the ability to perform important activities of daily living (4). Furthermore, participation in any enjoyable activity in a social setting is a cognitive activity that can stimulate cognition in people with mild-to-moderate dementia (5). This is important given that withdrawing from taking part in social activities is common for those living with dementia (6).

Older people who do not have individual strategies to overcome barriers to regular physical activity participation would probably need opportunities more tailored to their needs to be able to adhere (7). Strategies to engage individuals with cognitive challenges in exercise offers need to be innovative, especially knowing the beneficial role of the exercise suggested (8). PA guidelines promote the inclusion of strength and balance activities for older adults (2), and for those with cognitive impairment, the need for supervision and support is underlined (9). For people with dementia living at home, activities outside their homes can be challenging, due to the difficulties with participation (10). In one small study, the association between support after dementia diagnosis and social participation was explored (11), suggesting that the source of support is important. The authors of this study discuss whether stigma could be one explanation for decreased social participation and underline the need for appropriate support to contribute to a better living with dementia. In a comprehensive review of barriers and facilitators for PA in persons with dementia, several barriers were identified at the community level, such as transportation and difficulties finding their way (10). This is also in line with a more recent Norwegian study, where community-dwelling persons with dementia mentioned the loss of driver's license and dependency of others being a heightened threshold for participation in PA (12). Thus, convenient PA options with the necessary support are needed, also to avoid added burden on close informal caregivers (10).

People in early phases of dementia describe a need for both continuity of services and trained and educated professionals in dementia care (13). More person-centered care can lead to positive experiences that support people living with dementia in their everyday life (13). Positive experiences with making new friends and participating in meaningful activities are also reported as important for people with dementia living at home (13, 14). Caregivers may hold key roles for involving people with dementia in PA (10), but at the same time, they could experience a heavy caregiver burden already in early phases of dementia (15, 16). The support for caregivers during the disease process has been shown to be inadequate (17) and the burden of family caregivers is often overlooked (18). Thus, exercise activities for home-dwelling people with dementia should be established without such people being reliant only on close informal caregivers.

The World Health Organization (WHO) published a global action plan on the public health response to dementia for 2017–2025, where PA is highlighted as a modifiable risk factor for dementia (19). Further, in the World Alzheimer Report from Alzheimer's Disease international, PA interventions are supported as part of both dementia prevention strategies and an aim to maintain mobility, independence, and overall wellbeing (20). In Norway, the general policy is to support older people in their own homes (21). In the national Dementia Plan (22), the Norwegian authorities aim to create a more dementia-friendly society, which takes care of and integrates people with dementia into the community. To reach this goal, it will require greater openness and increased knowledge about dementia in society in general and in healthcare services in particular. In addition to knowledge development, areas of focus in the Dementia Plan are User Involvement and Participation, Prevention Services, and Public Health (22). To support the delivery of this plan, it is important to develop and evaluate PA programs that aim to support community-dwelling older people living with dementia. When creating new arenas for physical and social activities, it is important to ensure that people with dementia feel valued as individuals and are supported to maintain their identity (23). At the same time, we need to acknowledge the critical role played by family members who care for their older adults at home, and always create offers that can support their caregiving (24).

In a pilot, we developed and tested the first version of a new exercise concept in one municipality in Norway (25). The exercise content was developed based on an existing fall prevention exercise program for older adults (26), with volunteers from the municipality to support the participants both to get to and from the class, and during the exercise sessions (25). After making a more flexible model to facilitate implementation in diverse contexts, we started groups in three municipalities across Norway. Thus, in this study, we aimed to explore the experiences of participants from the existing exercise groups across the three municipalities.

## 2 Materials and methods

### 2.1 Study design

This study is part of a health project called *Still Active!* aimed to develop and implement group exercise sessions for people with cognitive impairment or dementia in three municipalities in Norway, and thus provide guidance to other municipalities on how to start such groups. In this qualitative study, using semistructured, in-depth, focus group interviews, we explored participants’ experiences of taking part in this program once a week over a 12-week period. This was a new concept, with the first exercise groups running from March 2021 to September 2021.

### 2.2 Development of the Still Active concept

The Still Active group exercise concept consists of the exercise content and the organization. The initial testing was organized by using volunteers recruited from the municipality and physiotherapists as instructors (25). We further developed the concept in collaboration with the original municipality and two new municipalities in Norway so that the concept could be generalizable. The content of the group exercise sessions was similar as in the first pilot, but the recruitment of volunteers was organized through the existing concept of “Activity Friends” (https://nasjonalforeningen.no/tilbud/aktivitetsvenn-for-personer-med-demens/fortsattaktiv/). For implementation purposes, the practical organization of the group sessions was tailored to each municipality. Table 1 presents the details about the participants, setting, and organization. Table 2 gives an overview of the volunteers involved in Still Active. Instructors, who were also volunteers, were recruited by the Physiotherapy Department in the three municipalities. Each exercise session was led by one or two instructors.

**Table 1:** Organization of Still Active exercise groups in the three municipalities.

Municipality	A (four participants)	B (three participants)	C (five participants)
Setting	Municipal wellness center	Municipal sports hall	Day care center in a nursing home
Content	Exercise + a short walk to a local café after the sessions where all could buy coffee and something to eat	Exercise	Exercise + a social event with coffee and a snack provided by the municipality
Reminders	Phone call from the municipality administrator	Phone call from volunteers	Phone call from the bus driver
Instructor(s)	One instructor responsible for the exercise sessions	Two instructors shared the responsibility for the exercise sessions	Two instructors shared the responsibility for the exercise session, supported by one volunteer
Activity friends	Four volunteers self-organized their support during the exercise and social coffee session after the exercise	Participants were matched, one to one, according to common interests. The three volunteers supported during both the exercise and the transportation	One Activity Friend supported the instructors during the exercise session and was responsible for the social event (preparing coffee and the snack). The Activity Friend also supported participants when arriving/leaving with the bus
Transport	Maxi-taxi with a dedicated driver trained in communication with persons with dementia. One of the volunteers joined the taxi	The activity friends were responsible for transportation one to one, from home to each session and back home, either by car or by walking	Municipal bus service with the same driver each time. The volunteer met the participants outside the day care center when arriving on the bus

**Table 2:** Volunteers in still active.

Activity friends	Instructors
Role: supporting the participants before, during, and after the exercise sessions	Role: planning and delivering the exercise sessions
Training: one-day course through the Norwegian Health Association's concept Activity Friend (*Fortsatt Aktiv!*) focusing on knowledge about dementia, communication, and the Activity Friends’ role	Training: training was twofold: 1. a 3-day course held by physiotherapists in their municipality through the Strong and Steady concept (21) focusing on knowledge about the ageing process, the instructors’ role, and “how to” do exercise to prevent functional decline and falls 2. an online version of the one-day course for Activity Friends (*Fortsatt Aktiv!*)
Follow-up: contact with the project volunteers’ coordinator when needed	Follow-up: regular meetings with the project physiotherapist discussing adaptations of the Strong and Steady exercises and how to be instructors for people with cognitive impairments, along with additional contact/support when needed

### 2.3 Participants

The target users of Still Active were home-dwelling people with cognitive impairment or dementia, who were able to walk independently without using walking aids and wanted to take part in a group exercise session once a week to maintain physical function. No formal testing of participants’ cognitive function was included in this study as we invited all persons who participated in the existing Still Active exercise groups in the three municipalities (A, B, and C). We included 12 out of 13 potential participants (*n* = 4 from group A, *n* = 3 from group B, and *n* = 5 from group C).

### 2.4 Interviews

We conducted focus group interviews immediately after completing one of the last exercise sessions of the first 12-week exercise period (in May 2021, June 2021, and September 2021).

We developed an interview guide based on our previous experiences from the pilot study (25) to guide the discussions in all interviews. We gave all participants time and opportunity to ask questions, both prior to starting and after ending the focus group discussions. We designed the questions as open-ended. The high-level topics used in the focus group interviews were: (1) motivation for taking part in Still Active, (2) overall experiences after the 12-week period, (3) experiences of being accompanied by an Activity Friend, (4) exercise sessions (content and instruction), (5) ideas for what to include in group exercise sessions such as Still Active in the future, and (6) what support is needed to be able to attend activities outside their home.

One or two interviewers from the research team (RG, AN, and KT) conducted the interviews with the participants on-site. Because of the target population being persons with cognitive impairment or dementia, the Activity Friends were present during the interviews in municipalities B and C. Furthermore, experienced researchers with a clinical background, who were not involved in the delivery of the group exercise program, facilitated the discussions. The interviews lasted between 30 and 45 min.

### 2.5 Data analysis and reflexivity

All interviews were audio-recorded and transcribed verbatim by experienced researchers (KT, AN, and RG). The audio-recorded data were checked and compared with the transcripts by a second researcher prior to any analysis. We used a systematic text condensation method, inspired by Malterud (27), for data analysis. As a first step, three of the authors read the transcripts independently to get a general sense of the data set. After discussing the overall impression, the next step was to identify meaning units. We defined meaning units as text fragments reflecting information about the participants’ experiences with attending the volunteer-supported group exercise program. The authors then started identifying and sorting meaning units into codes. During this phase, the authors reflected on the similarities and differences of each code. Based on a consensus after reading the transcripts of all three interviews, three authors (KT, AN, and RG) agreed on the final codes. The first and the last author coded the interviews into final concepts and discussed them before they were presented and also discussed them with a wider group of coauthors (EB and GT). The three authors involved in the data analysis are all physiotherapists by background, with varying experience. We ensured the trustworthiness of the data through a final check with the whole group of coauthors, including the four coauthors involved in the organization of the exercise offer in the municipalities (GG, ME, NW, and LJ).

### 2.6 Ethics

The Regional Committee for Medical Ethics South-East Norway (REK 2019/65784) confirmed that the study did not need their approval. The Norwegian Centre for Research Data (NSD) approved the study in 2020 (Ref. No. 421413). Information about the focus group interviews was given to all participants orally at the start of this study and repeated orally before the focus group interviews. Participants signed an informed consent form prior to the interviews.

## 3 Results

**Figure 1:**
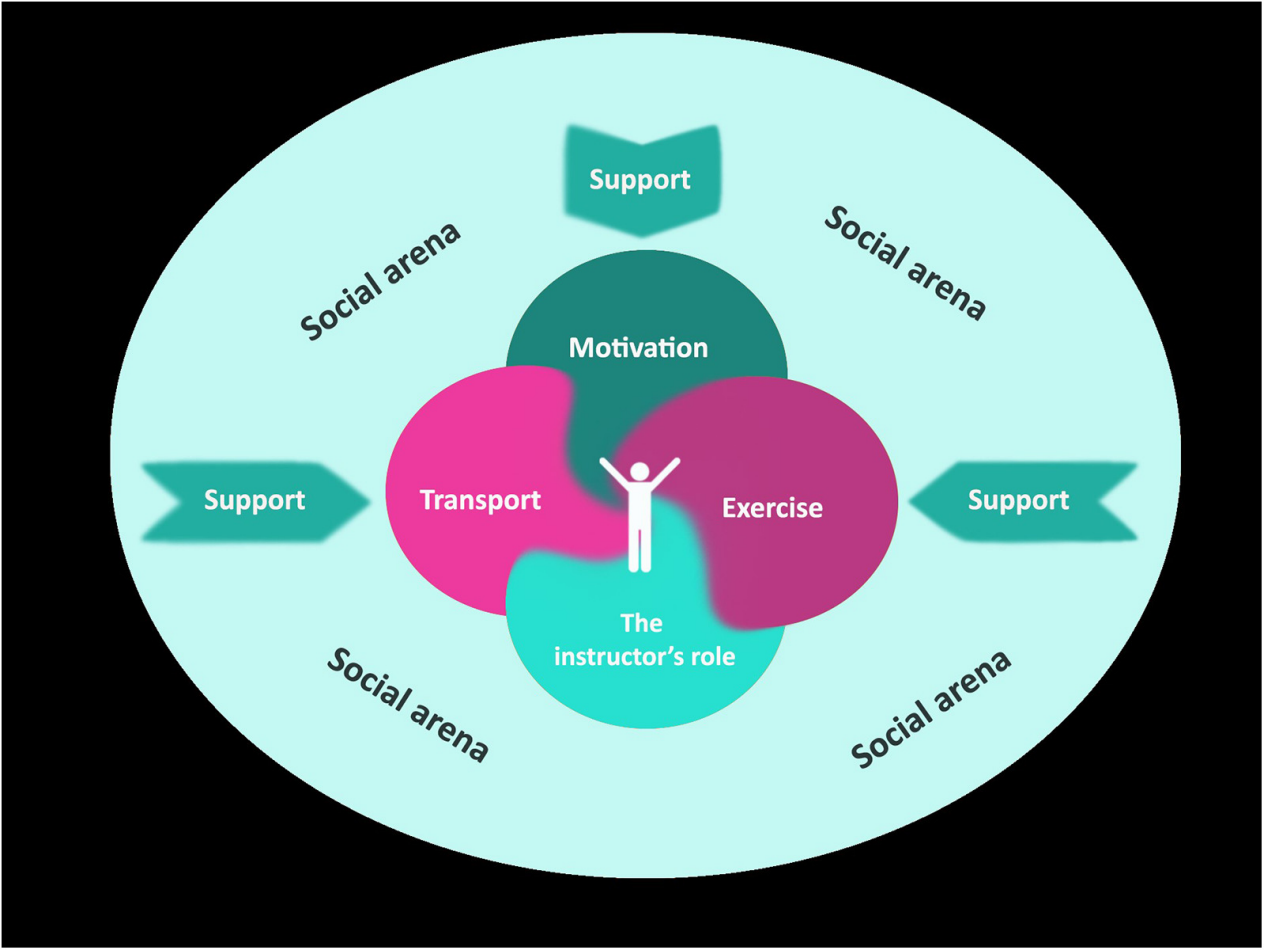
Overarching concepts and subconcepts experienced by the participants in Still Active.

We included four women (69–78 years) and eight men (72–82 years), of whom one lived alone. The three focus group interviews with the 12 participants demonstrate that for older adults with dementia, participation in group activities outside their home is highly dependent on support. The themes reflect four elements of support that are essential for the participants to be able to take part in the group exercise offers, and a fifth theme describing group exercise as their social arena. The five themes were as follows: (1) motivation for taking part in activities outside the home, (2) transportation, (3) exercise content, (4) the instructoŕs role, and (5) having a social arena. The themes are illustrated in Figure 1 and Table 3.

**Table 3:** Presentation of key themes, summary, and quotes.

Key themes	Summary	Quotes
Motivation for taking part in activities outside home—*He encourages me*	A trusted person (caregiver, healthcare professional, or volunteer) played a key role in encouraging participation. Participants were sometimes unsure why they joined but were motivated by external support. Ultimately, participants recognized their own decision to take part	“It was my wife … she encourages me.” “I am not quite sure why I am here, but my wife signed me up …” “It was the physiotherapist … she came to our house several times, talked to me, and made me do some exercises”
Transportation—*The transport which is fantastic*	Essential for participation as none of the participants could drive. Provided a sense of safety, convenience, and social connection. Different transport arrangements across municipalities: Municipality B: Transport by an Activity Friend, creating a close bond. Municipality A and C: Minibus/maxi-taxi service, with engaging and friendly drivers	“… had probably not managed to come to the group session without the transport” “He [the driver] has humor and is always in a good mood.” “The transport … feels like we have become siblings”
Exercise Content—*I feel happy when I am exercising*	Participants appreciated structured physical activities. Some had prior experience with exercise programs; others had limited opportunities. They found the exercises important and motivating as it felt good. Three men discussed the benefits of structured weight training. Overall, the focus was on maintaining function, preventing decline, and enjoyment	“I didn't want to sit down to talk and talk and talk …” “If you give up, it is over …” “… it has been so enjoyable …”
Instructor's Role—*They can see people*	Participants highly valued instructors who were attentive and engaging. Instructors were praised for knowing participants by name, adapting exercises, and fostering a welcoming atmosphere. Balance exercises were particularly appreciated as both challenging and necessary	“… they [the instructors] know us … use our names.” “The instructor … observe if we struggle … and adapts some of the exercises.” “Have a feeling that she is like us”
Social Aspects—*To meet people and become familiar with them*	Social interaction was just as important as exercise. Participants formed friendships through transportation and exercise groups. Additional social gatherings (e.g., coffee sessions) were highly valued	“It's the social part … and then I also like to move a little bit.” “… exercise … it is social … this helps a lot in the everyday life…” “… nice closure”

### 3.1 Motivation for taking part in activities outside home—he encourages me

All participants experienced motivation from *someone* as crucial for them to be able to participate in activities, especially when starting a new activity. This “someone” was a trusted person from whom the participants took advice. This person was either their closest informal caregiver/next of kin, an employee from the healthcare system (general practitioner, physiotherapist, staff person at a day care center, and a dementia coordinator), or a close volunteer. The role of this person was, according to the participants, both to provide information about the exercise program, to warmly promote joining this activity, and then motivate them to take part.

One of them expressed as follows: “It was my wife … and I thought it sounded interesting … my wife, who reviews all papers, had seen an information sheet about this new group exercise session … she encourages me” (A, I3). One other who was already supported by an Activity Friend, expressed as follows: “It was X [the volunteer friend] who was so generous with me … he encourages me” (B, I7).

Interestingly, some of the participants highlighted that they were unsure why they had joined this group, but that support from someone made them join. As one informant said: “I am not quite sure why I am here, but my wife signed me up (for this group)…” (C, I12). Another informant said:

“It was the physiotherapist, I don't remember her name … I don't really understand why, but she came to our house several times, talked to me, and made me do some exercises … I was a bit surprised because I thought I was going to join another group exercise called Strong and Steady, but then she called me after her visit and said that it would be better for me to join this group … and she was right, because of the transport which is fantastic” (A, I4).

Although they all talked about the “someone” who informed, inspired, and motivated them to take part, and not all were quite sure why, all agreed that it had been their own decision to participate in the exercise group. As one of the informants said: “I think it was myself … yes … It was I—myself who decided that I wanted to join (this group)” (A, I1).

### 3.2 Transportation—the transport that is fantastic

All participants emphasized the transportation from their home to the activity and the return home as crucial for their participation. None of them were now allowed to drive a car, and some expressed that they missed being able to do so. Being transported by someone was important for them all. This was illustrated by one of the informants saying that he would give 50% of the value of the group exercise sessions to the transportation, as he “… had probably not managed to come to the group session without the transport” (A, I4).

The transportation was part of the Still Active program, and although the transportation was organized differently in the three municipalities, all participants described the feeling of safety and being together. In municipality B, the matched Activity Friends were responsible for the transportation to and from the class, and the participants told us that they experienced it as a good social moment with their special friend, and all of them expressed satisfaction with this way of organizing the transportation to the exercise group. One informant put it like this:

“It is positive that I have my own person who is coming to pick me up and reminds me that I am going to the exercise group … then I think, that's okay. You do not do any other things you know… After a while the car has arrived … and (as you know) … I am not allowed to drive a car anymore” (B, I7).

In municipalities A and C, the participants were offered transportation in the form a minibus or a maxi-taxi. In municipality C, the municipality's own bus picked up all but two of the participants, and they all expressed that they were satisfied with the transport, especially the bus driver. They said that he reminded them before picking them up and also arranged the transport. According to them, he also created a very good atmosphere in the bus on their way to the exercise session. They all talked about the driver as not an ordinary driver … and said: “he has humor and is always in good mood. He is talking … and has a lot of humor …” (C, I11).

The group sessions were not located close to all participants’ homes and transportation was necessary, and four of the participants in municipality C highlighted the social aspect of the bus trip. They said: “Very nice, you know, being picked up and driving with the bus here and there, and drive by and see where the others live….” (C, I12) and “Good to get a sightseeing in the morning” (C, I8).

Municipality A used a maxi-taxi in which a dementia-trained taxi driver picked up all participants at their homes and returned them home again. The participants lived in different parts of the municipality, which resulted in quite a long driving distance for some of them. Nevertheless, all participants reported high satisfaction, both with the safe transport and with the characteristics of this special taxi driver. Some of them mentioned the group as a new family. One of them said: “It starts with the drive … it's fantastic … the transport … It feels like we have become siblings” (A, I3).

### 3.3 Exercise content—I feel happy when I am exercising

All participants talked about their interest in being physically active, wherein some talked about earlier experiences with organized exercise. In municipality B and C, most had other offers outside their homes, in terms of day care offers, with municipality C also offering a session of physical exercise lead by a physiotherapist twice a week. None of the participants in municipality A had other physical or social activities outside their homes, as one woman said:

“I was participating in an exercise group, arranged by the health service … and when they informed me that they would turn this offer into a talk group, I decided not to take part … I didn't want to sit down to talk and talk and talk … so I told them that I wanted to quit. After my decision one of them asked me if I wanted to take part in this new exercise group” (A, I2).

Most of the participants expressed that doing exercise was motivating and that they felt good: “To use your body in a different manner. Yes.” (B, I5) and “I can still continue to do such exercise, I think it has been so enjoyable, and I think I feel that it has been good for my body” (B, I5). Two others said: “…perhaps to maintain functioning…” (A, I2) and “… yes, that we maintain/stop the process. If you give up, it is over, you know, and then you will just be sitting there, gasping” (A, I3). Others did not express it so clearly, but highlighted the enjoyment, as illustrated with this quote: “I want to go every time, and when I know I must go I want to go” (B, I7).

In one of the groups, three men, who previously had joined a similar exercise group, discussed weight training vs. light exercise. The group they used to be part of was led by a physiotherapist, with weight training as part of the program. As they discussed:

(C, I9) “I also have to say, that I think this is kind of light exercise, so to say, but it must be as part of a group….” (C, I10) “Yes. Perhaps a bit lighter now, but it is anyhow exercise, and a lot of what we do is similar from session to session, but what is more important is that we attend this exercise and I feel that it is pretty ok. For me, it is ok.” (C, I12) “It is kind of ordinary exercise I would say.”

### 3.4 The instructor's role—they can see people

All participants described the instructors’ role as of great importance. They explained that the instructor must be insightful about people and be able to see the person and show interest in the individual participant. As two of the participants discussed:

“‘a person with an understanding of character, I think it is very important that they can see people (A, I1) Interviewer: In what way/how do you feel that they see you? (A, I1): Yes, I feel… (A, I3 and I1 together) they know us. Our names, they use our names. Everyone does that.’ One of the same participants said: ‘They see me!’ (A, I1). Another one expresses it like this: ‘The instructor uses our names, is watching us and can observe if we struggle, and if so, she facilitates and adapts some of the exercises’” (A, I3).

Concerning the exercise session, all participants agreed that the instructor must be clear when they demonstrate the exercises. Several of them expressed the importance of having fun when exercising, but in addition, the exercise had to be challenging, especially when it came to the balance training.

All participants said they liked exercising and were satisfied with the exercise sessions, and they liked the selection of exercises that most were familiar with from before. As one of the women participants said: “I like exercising … and it reminds me of the gymnastics at school … I was working in the school … and this exercise is quite similar” (A, I2). Most of them highlighted the balance tasks as challenging, but really liked this. At the same time, they talked warmly about not focusing on performance, and that they felt safe during exercising. Furthermore, they expressed that they really enjoyed taking part, as one woman said: “I don't know what to say… I feel happy when I am exercising” (A, I2).

The participants experienced the feeling that the instructors were like one of them, as one said: “Have a feeling that she is like us” (A, I2). And they continued explaining: “We are able to talk with each other” (A, I3).

### 3.5 Having a social arena—to meet people, and to become familiar with them

Overall, all participants expressed that having a social arena was important to them. As one of the women said: “It is specially the social part … and then I also like to move a little bit… the exercising is very pleasurable” (B, I6). The man in this group expressed that he felt “pleasure” when participating in such a group and said, “I don't push it away, no I continue” (B, I7). Another expressed as follows: “Some exercise and some … something social … To meet people, and to become familiar with them” (C, I10). Furthermore, one man expressed that he needed to be more active, and that “… hiking alone was not the same. When it rained, I did not go outside …. Probably, if I thought it would rain, I also would not go outside” (C, I9).

Some of the participants referred to the other participants as their new friends or family members. These new friendships began with the transportation when they were on their way to the exercise session. Most of them talked about opportunities to get to know other people by joining this new exercise program, and that these people have now become their new friends. As one explained:

“In addition to exercising, it's also the social part. We all know each other now and are a group of friends” (A, I3). One other participant explained: “As a retired person, you know, it is not much to do, I am afraid of becoming passive, and being able to join such a group is great. Firstly, you get some exercise, secondly, it is social, and I think that this helps a lot in the everyday life for us as retired persons. Yes, perhaps it was what I wanted. Some exercise and some, yes, some social time” (C, I10).

Time to talk and have a coffee and something to eat was added as part of the groups in municipalities A and C. The participants appreciated this social meeting. In one of the groups, they explained that the social session was enjoyable as well as a good way to end the exercise sessions, as they said, “It is very positive” (A, I2), “… nice closure” (A, I1 and I3), and “then we get to know each other” (A, I2) “… talk more with each other” (A, I3). The Activity Friends had a clear role in the social part in these two groups. For the municipality where there was only one Activity Friend for the whole group of participants, the social part was organized in the same building as the exercise session. The participants said: “She is organizing coffee and some food after the exercise” (C, I10) and “she fixes all the practical things, and we just ask her, and she sort it out” (C, I11).

## 4 Discussion

In this study, we explored experiences from home-dwelling, older adult participants, with cognitive impairment or dementia, who took part in a newly developed PA program supported by volunteers. All participants expressed satisfaction with Still Active, as they had the opportunity to be part of an exercise group and got to know and to talk to other people outside their closest family. All had stopped participating in previous activities, even though they all had positive experiences with PA and exercising in the past. They expressed a wish to be more physically active and to continue exercising.

Our participants underlined the need for support to be able to take part in PA outside their home, which is in line with what has been reported in previous studies (9, 10). We found that the social arena is not only present in the group exercise sessions, but also significant for the participants during the implementation of the whole Still Active concept. The social aspect starts and ends with the person who picks them up and drives them back home, whether this is the one-to-one Activity Friend or the taxi- or bus driver. Overall, having a social arena was highlighted as important by the participants from all three municipalities. For people who have stopped many of their previous activities, and where they are being more restricted to stay in their homes, having their own social arena seems to be valued. This is in line with previous research, describing a need for meaningful activities delivered in environments where people living with dementia can feel respected and valued (16). Our results show that several of the participants referred to the other participants as their new friends, a description that was similar to what we found in the pilot study (25). Having a social arena where people could have fun and feel valued and respected could help in maintaining their identity and in providing new experiences (23).

For people with cognitive challenges, the combination of structured activity and people who understand is suggested as an important facilitator for undertaking PA (28). All participants but one lived together with their spouses, who they said was their main support person. Several studies have pointed out that informal caregivers to people living with dementia consider it a burden to being their main support person (29, 30), and that social networks and friends often disappear (31). Having a social arena outside their homes could thus be crucial for both the person with dementia and their spouse or caregiver. For people living with dementia, positive experiences gained from other settings also gives them the opportunity to tell their own stories when they come back home (23).

To be able to be socially active and to take part in such groups outside their homes, support in all aspects was highlighted by the participants as crucial. From the literature, we know that caregiver burden is high and thus offers with support provided from others are warranted (18, 28, 32). Our group exercise sessions provided an arena where the participants could attend with support from individuals from the time they were picked up to the time they were taken back home. To be able to have dementia-friendly communities, strategies that support people living with dementia in communities should include the role of individuals in many different roles (33).

Motivation to engage in activities would be the first barrier when accepting new offers. Our participants said they needed support from someone to be willing to attend. One other study also highlighted that support to engage in the community in this population is important (33). In a previous study, we found that caregivers are willing to promote offers if they are considered meaningful (29). One interesting result of the current study was that all participants in our three groups wanted to continue, describing the group exercise sessions as positive overall. Motivation was only needed to be able to accept and engage in the sessions, but after starting, they all wanted to continue.

Our group exercise sessions provided transport to and from the exercise sessions, and participants described this facility as fantastic. Organized transport made it possible and easy for them to attend, and they highlighted the person providing the transport as important. Having the same driver every time was valued, and they really liked being picked up and having a great time going to the exercise sessions. This was also true for the participants who went with their Activity Friend, confirming that all parts of these sessions should be facilitated by people and organizations that understand the unique needs of this group (28). None of the participants drove a car anymore and said they considered losing their drivers’ licenses a big loss. The importance of transportation has been reported earlier, and one study found that transportation played a role in limiting people with cognitive impairments, more than healthy individuals, (34) from going out for enjoyment.

For the participants, it was important that the sessions involved exercise as they considered this more meaningful than just social activities or conversation groups. Exercise in itself and the positive effects of exercise motivated them to take part in Still Active. This is in line with previous research showing the importance of motivation for engagement in sessions also for persons with dementia (35). In addition, the way the exercise is presented is of great importance to the participants, which leads us to the instructors’ role. The importance of the exercise instructors is supported by a scoping review that found the instructor is regarded as crucial both for the social environment and for the content in an exercise group (36). According to the review, an instructor should be able to understand group dynamics and be a good communicator. Furthermore, older adults may favor instructors who are like themselves, because this makes them more relatable. For the content of an exercise group, the competence of the instructor is important, whereas knowledge about both exercise and gerontology are valued (36). This is in line with our findings, where our informants explained that the instructor must be insightful about people and able to see people. Instructors in Still Active were trained volunteers, with continuous follow-up from municipality physiotherapists.

Overall, the support from individuals, in different parts of group exercise sessions, seems to be important for people with cognitive impairment or dementia. It enables them to take part and to be socially active in an activity, which is important for maintaining function by being physically active. In the Still Active sessions, support was provided by someone who made them feel safe, who they described as new friends, underlining the importance of the people involved in such group exercise sessions. One study in which exercise providers were included showed that, although being willing, there is a need for knowledge to be able to create a dementia-inclusive exercise (37), and our study provides one suggestion for how to run such an exercise offer in a municipality setting. The Still Active concept could enhance the wellbeing, inclusivity, and accessibility of exercise for older adults with impaired cognition or dementia in the community, and thus contribute to creating a more inclusive environment aligning with the principles of age-friendly cities (38).

The major strength of this study is that we included the voices of people with cognitive impairment or dementia. The Still Active concept was tailored by the use of two steps, first on an overall level to the target population, and second, to the local setting in each municipality. Another major strength is that we conducted the study across three municipalities and included participants from all three sites. Our participants described exercise as very positive and all had previous experiences of either being physically active or attending organized exercise, and thus our results cannot be transferred to other groups who do not find exercise meaningful. We acknowledge that a sample of 12 participants is small and that the setting is limited to one Nordic country. One negative aspect of the study is that although our participants felt an overall positive experience with the new group exercise sessions, this could have been dictated by the apprehension that they could lose these exercise sessions and that this probably was one out of very few activities they had outside their homes. Furthermore, we did not include a control group, and we do not know whether a similar social group would have provided the same experiences. The study was conducted at the end of a 12-week exercise period, and experiences with participation over a prolonged period in this target population would also have been of interest. Nevertheless, we believe that the study results raised important points that should be included for future design and delivery of similar group sessions.

## Conclusion

In this study, we examined the experiences of older adults with cognitive impairment or dementia who participated in group exercises supported by the use of volunteers, finding them to be highly satisfied with group exercise sessions outside their homes especially as this creates a social arena for them. Although creating high-quality content in such groups is important, support is evaluated as crucial to be able to both accept and attend such group sessions outside their homes. Implementation strategies for exercise sessions should focus more on organization strategies in the future. Individuals who provide support, ranging from recommending the offer and assisting with transportation to facilitating the exercises, play a crucial role for older adults with cognitive impairments, as the effectiveness of exercise is highly dependent on support at every stage.

## Data Availability

The datasets presented in this article are not readily available because we do not have approval from the NSD nor the participants to share data. Requests to access the datasets should be directed to kristin.taraldsen@oslomet.no.
